# Construction of a High-Density Genetic Map and Identification of Quantitative Trait Loci for Nitrite Tolerance in the Pacific White Shrimp (*Litopenaeus vannamei*)

**DOI:** 10.3389/fgene.2020.571880

**Published:** 2020-09-24

**Authors:** Min Peng, Digang Zeng, Weilin Zhu, Xiuli Chen, Chunling Yang, Qingyun Liu, Qiangyong Li, Huanling Wang, Hong Liu, Jingzhen Liang, Yong Lin, Xiaohan Chen, Yongzhen Zhao

**Affiliations:** ^1^Guangxi Key Laboratory of Aquatic Genetic Breeding and Healthy Aquaculture, Guangxi Academy of Fishery Sciences, Nanning, China; ^2^Key Lab of Freshwater Animal Breeding, Key Laboratory of Agricultural Animal Genetics, Breeding and Reproduction, Ministry of Education, College of Fishery, Huazhong Agriculture University, Wuhan, China; ^3^Life Science Research Institute, Guangxi University, Nanning, China

**Keywords:** genetic map, QTL, transcriptomic, nitrite tolerance, *Litopenaeus vannamei*

## Abstract

Nitrite is a major environmental toxin in aquaculture systems that disrupts multiple physiological functions in aquatic animals. Although nitrite tolerance in shrimp is closely related to successful industrial production, few genetic studies of this trait are available. In this study, we constructed a high-density genetic map of *Litopenaeus vannamei* with 17,242 single nucleotide polymorphism markers spanning 6,828.06 centimorgans (cM), with an average distance of 0.4 cM between adjacent markers on 44 linkage groups (LGs). Using this genetic map, we identified two markers associated with nitrite tolerance. We then sequenced the transcriptomes of the most nitrite-tolerant and nitrite-sensitive individuals from each of four genetically distinct *L. vannamei* families (LV-I–4). We found 2,002, 1,983, 1,954, and 1,867 differentially expressed genes in families LV-1, LV-2, LV-3, and LV-4, respectively. By integrating QTL and transcriptomics analyses, we identified a candidate gene associated with nitrite tolerance. This gene was annotated as solute carrier family 26 member 6 (*SLC26A6*). RNA interference (RNAi) analysis demonstrated that *SLC26A6* was critical for nitrite tolerance in *L. vannamei*. The present study increases our understanding of the molecular mechanisms underlying nitrite tolerance in shrimp and provides a basis for molecular-marker-assisted shrimp breeding.

## Introduction

The Pacific white shrimp (*Litopenaeus vannamei*) is the world’s most extensively cultivated shrimp species (Lu et al., 2016; Javahery et al., 2019); annual production of *L. vannamei* accounts for about 50% of all penaeid shrimp production (Xiao et al., 2019). In 2016, the global production of *L. vannamei* approached 4.16 million tons (Xiao et al., 2019). However, the intensive or semi-intensive farming model commonly used at present for shrimp aquaculture often leads to water quality deterioration, and the resulting toxicological stress causes mortality or disease in farmed shrimp, negatively affecting breeding efficiency (Sun et al., 2015).

Nitrite, a strong oxidant that is the intermediate product of the biological oxidation of ammonia to nitrate (Valencia-Castaneda et al., 2019), is one of the most common toxins in aquaculture water (da Silva et al., 2018). Exposure to nitrite decreases oxyhemoglobin levels in mammals, leading tissue hypoxemia and hypoxia (Cheng and Chen, 2002b; Kim et al., 2019). As the prosthetic group of the hemocyanin in crustacean blood is also a copper-containing compound, hemocyanin may behave similarly to hemoglobin; therefore, nitrite exposure in crustaceans might lead to hypoxia, cyanosis, or even death (Chen and Lee, 1997; Cheng and Chen, 2002a). Chen and Lei reported that the 96-h LC_50_ of nitrite was 3.91 mM on *P. monodon* (Chen and Lei, 1990). Working on seven species of penaeid shrimps, Wickins reported that the 48-h LC_50_ of nitrite was 12.1 mM (Wickins, 1976). The destructive effect of nitrite on the oxygen-carrying capacity of hemocyanin is considered one of the main mechanisms of nitrate toxicity in shrimp (Jiann-Chu and Cheng, 1995). Numerous studies have shown that in nitrite-stressed shrimp, nitrite concentrations in the hemolymph increase, while levels of oxygen-binding hemocyanin, total protein, and hemolymph oxygen affinity decrease significantly (Chen and Cheng, 1995; Jiann-Chu and Cheng, 1995; Sha-Yen and Chen, 1999; Cheng and Chen, 2002b). Hemocyanin is an oxygen-carrying protein in crustaceans (Giomi and Beltramini, 2007). After nitrite enters the hemolymph, it causes the conversion of oxygenated hemocyanin to deoxyhemocyanin, and inhibits the binding of deoxyhemocyanin to oxygen (Cheng et al., 2013; Li et al., 2019). In addition, similar to the mechanism by which nitrite destroys mammal hemoglobin, nitrite may further cause the oxidation of deoxyhemocyanin to denatured hemocyanin (Cheng et al., 2013; Li et al., 2019). Studies have shown that nitrite reacts 15 times faster with deoxyhemocyanin than with oxyhemocyanin (Tahon et al., 1988). As a result, nitrite causes a decrease in the oxygen affinity of the shrimp hemolymph, eliminating its normal oxygen carrying capacity, and eventually resulting in asphyxiation (Jiann-Chu and Cheng, 1995). Indeed, it has been reported that nitrite has serious deleterious effects on shrimp growth (Wasielesky et al., 2017), immunity (Tseng and Chen, 2004), and survival (Wasielesky et al., 2017; Valencia-Castaneda et al., 2018, 2019). Therefore, low aquatic nitrite concentrations must be maintained for successful shrimp farming. However, aquaculture water quality is affected by many factors, including weather and artificial feeding (Paez-Osuna, 2001), and efforts to control nitrite levels in aquaculture water are not always effective. Although the establishment of new, nitrite-tolerant shrimp varieties might represent an effective solution to this problem, such breeding programs require a prior understanding of the genetic basis of nitrite tolerance in shrimp. Unfortunately, the genetic basis of nitrite tolerance in shrimp has yet to be characterized.

Genetic maps play an important role in the identification of quantitative trait loci (QTLs) and functional genes associated with economically valuable traits in plants and animals (Zhu et al., 2016). In addition, genetic maps can be used to assemble sequences for genome sequencing, genome structure comparison, and marker-assisted selection (MAS) (Wang et al., 2011; Okuda et al., 2012; Jones et al., 2013). Previously, genetic map construction was primarily based on traditional molecular marker techniques (Kuiper, 1998; Duran et al., 2004; Maughan et al., 2004). The fusion of second-generation sequencing technologies with genotyping methods, including genotyping-by-sequencing (GBS) (Poland et al., 2012), restriction site-associated DNA sequencing (RAD-seq) (Peterson et al., 2012), and specific length amplified fragment sequencing (SLAF-seq), allow the identification of millions of single nucleotide polymorphisms (SNPs) in plant and animal genomes. In addition, these technologies produce markers that are denser, more consistent, more efficient, and less costly than traditional methods (Sun et al., 2013).

To understand the genetic mechanisms underlying nitrite tolerance in shrimp, and to identify related candidate genes, we constructed a high-density SNP genetic map of *L. vannamei* using SLAF-seq. Using this genetic map in conjunction with phenotypic data (survival time under acute nitrite stress), we identified QTLs for nitrite tolerance in *L. vannamei*. We further compared transcriptomic differences between nitrite-tolerant and nitrite-sensitive *L. vannamei* from genetically distinct families using RNA sequencing (RNA-seq). We then performed a comprehensive analysis of gene expression profiles and QTLs to identify candidate genes associated with nitrite tolerance. Finally, we verified candidate genes using RNA interference (RNAi).

## Materials and Methods

### Establishment of the Mapping Family

The *L. vannamei* family used for mapping was established at the National and Guangxi Shrimp Genetic Breeding Center (Guangxi Province, China). To develop the mapping family, the male parent was selected from a family with relatively high nitrate tolerance, and the female parent was selected from a family with normal nitrate tolerance. To identify families with different levels of nitrate tolerance, 20 families were exposed to nitrate (700 mg/L) for 3 days. We considered the family with the highest survival rate to have a relatively high level of nitrate tolerance, and the family with the lowest survival rate to have a normal level of nitrate tolerance. We then exposed 40 shrimp from the relatively high tolerance family to 700 mg/L nitrite for three days, and randomly selected the male parent from the five surviving shrimp. The female parent was selected randomly from the family with average nitrate tolerance. The parent shrimp were artificially inseminated, and their progeny were cultured in a pond. After cultivation for one year, a male shrimp and a female shrimp were randomly selected from the progeny and artificially inseminated. Their progeny (the F2 population) were used as the mapping family (LV-1).

### Acute Nitrite Stress Test

We randomly selected 157 shrimp from the mapping family (8-months-old; average mass: 20.46 g). Selected shrimp were cultured in a 2 × 4 × 1 m indoor pool. The pool water remained aerated throughout the acclimation period and the experiment, with pH maintained at 8.2 ± 0.3; temperature maintained at 27.0 ± 0.5°C; salinity maintained at 30.1‰; and dissolved oxygen maintained at 7–8 mg/L. All shrimp were fed formulated pellets (Zhengda Corporation, China) daily at a ratio of 5% of average body weight. Shrimp were allowed to acclimate for 5 days before the acute nitrite stress test. Analytically pure NaNO_2_ was dissolved in filtered seawater to prepare a concentrated nitrite stock solution; this stock solution was then added to the pool water to increase the nitrite concentration to 700 mg/L. This concentration was chosen, because preliminary experiments (Supplementary Table S1), all experimental shrimp died within 80 h at a nitrite concentration of 700 mg/L, and this time range was suitable for observation. The nitrite concentration in the pool was measured every 24 h using the standard method (Gilcreas, 1967). Filtered seawater or nitrite stock solution was added to the pool as needed to maintain the nitrite concentration at 700 mg/L. Dead shrimp were collected every 1 h. Shrimp were considered dead when lying on the bottom of the pool out of balance and unreactive when touched with a wooden stick. Survival time was recorded to represent nitrite tolerance. Dead shrimp were collected immediately upon observation, frozen in liquid nitrogen, and transferred to an ultra-low temperature freezer (−80°C) until DNA extraction.

### DNA Extraction, SLAF Library Preparation, and Sequencing

We extracted DNA from the tail muscles of the 157 selected F2 progeny and the two parents using Marine Animal DNA Extraction kits (Tiangen, China). The quality and quantity of each extracted DNA sample was measured using 1% agarose gel electrophoresis and a ND2000 spectrophotometer (NanoDrop, United States), respectively. We used restriction enzyme digestion prediction software (Biomarker Technologies Corporation, China) to predict appropriate endonucleases for genomic fragmentation based on the genome of *L. vannamei*^1^ (Zhang et al., 2019). Based on this prediction, all genomic DNA samples were digested with the enzymes *Hae*III and Hpy166II. The dual-index adapter was then ligated to the fragments using T4 ligase, and polymerase chain reactions (PCRs) were performed. Amplification products (314–414 bp, including the adapter) were purified and collected using gel extraction kits (Illumina, United States). These fragments were the re-amplified for SLAF sequencing using PCR. SLAF sequencing was performed using an Illumina HiSeq system (Illumina, United States), following the manufacturer’s instructions. To evaluate the accuracy of library construction, we used the same procedures to construct SLAF sequence libraries for *Oryza sativa L. japonica* as a control. All library construction and sequencing were performed at Biomarker Technologies Corporation (Beijing, China).

### SLAF-Seq Data Analysis and Genotyping

First, the raw sequencing data were assigned to samples based on the dual-index adapters. These adapter sequences were removed, and the reads were filtered to remove all reads containing the adapter sequence, as well as reads containing more than 10% unknown (N) bases. As the first few bp of each read were the residue left by the enzyme fragment, the sequencing quality in this area was low. Therefore, only bases 4–103 bp were analyzed (fragment length: 100 bp). The filtered clean reads were compared to the *L. vannamei* genome^1^ (Zhang et al., 2019) using BWA software (Li and Durbin, 2009). Reads with >95% identity, and where both ends matched to the same location in the *L. vannamei* genome, were considered derived from the same SLAF marker. SNP-based polymorphic SLAF markers were identified by comparing reads derived from the same SLAF marker. These polymorphic SLAF markers were filtered to remove markers whose parental sequencing depth was less than 10×; markers with >5 SNPs; markers for which the proportion of genotypes covering all offspring was <70%; and markers with severe partial segregation (chi-squared test *P*-value < 0.05). The remaining polymorphic SLAF markers were encoded into eight segregation patterns: ab × cd; ef × eg; hk × hk; lm × ll; nn × np; aa × bb; ab × cc; and cc × ab. As the mapping population we used was an F2 population, the polymorphic SLAF markers with the aa × bb separation pattern were discarded, and the remaining polymorphic SLAF markers were used to construct the linkage map.

### Genetic Map Construction and QTL Analysis

The genetic map was constructed with iterative ordering and error correction, as implemented in HighMap (Liu et al., 2014). The polymorphic SLAF markers were imported into HighMap, and the linkage groups (LGs) were determined using the pair-wise logarithm of the odds (LOD) test. The order of the markers in the LG was calculated using the enhanced Gibbs sampling, spatial sampling, and simulated annealing (GSS) algorithm (Liu et al., 2014). The genetic distances between markers were calculated in centiMorgans (cM) based on recombination values using the Kosambi function (Kosambi, 1944), which has been widely used in association studies and has the advantages of reflecting adequate levels of double recombination in species populations (Ju et al., 2008). QTL analysis was performed using R/qtl software package (Broman et al., 2019). The LOD threshold for each data set was established based on the permutation test (1,000 permutations; *P* < 0.05). QTLs with LOD values above this threshold were considered significant. The phenotypic variance explained (PVE) by the two QTLs was estimated using the following formula: 1 − 10^–2LOD/^*^*n*^* (Gardiner et al., 2012), where *n* was the sample size.

### RNA-Seq

RNA-seq analyses were performed using shrimp from four families: the mapping family (LV-1) and three additional genetically distinct families (LV-2, LV-3, and LV-4). Our previous analysis indicated that the 24-h median lethal concentration (LC50) of NaNO_2_ was 194.6, 196.3, 174.3, and 250.8 mg/L for families LV-1, LV-2, LV-3, and LV-4, respectively (Supplementary Table S2).

We randomly selected 200 shrimp from each family. Each family group was subjected to the acute nitrite stress test (700 mg/L nitrite), as described above with minor modifications. Briefly, in each family group, we collected the 20 most nitrite-sensitive shrimp (i.e., the shrimp with the shortest survival times) and the 20 most nitrite-tolerant shrimp (i.e., the shrimp with the longest survival times). Nitrite sensitive shrimp were collected as soon as soon as they began to exhibit symptoms of toxicity (i.e., swimming out of balance). The hepatopancreases were removed from all collected shrimp and pooled to generate nitrite-sensitive and nitrite-tolerant pooled samples for each family. After removal and pooling, hepatopancreas tissues were immediately frozen in liquid nitrogen and stored in an ultra-low temperature freezer (−80°C) until RNA extraction.

Total RNA was extracted from each pooled sample using TRIzol reagent (Invitrogen, United States), following the manufacturer’s instructions. Residual genomic DNA was removed with DNase I (Invitrogen, United States). RNA integrity was evaluated using the Agilent Bioanalyzer 2100 system (Agilent Technologies, United States), and RNA concentration was measured using a ND2000 spectrophotometer (NanoDrop, United States). Sequencing libraries were constructed using NEBNext UltraTM RNA Library Prep Kits for Illumina (NEB, United States), following the manufacturer’s instructions. The libraries were sequenced on an Illumina HiSeq 2500 platform and paired-end reads were generated. Library construction and sequencing were performed at Biomarker Technologies Corporation (Beijing, China).

Raw sequencing reads were cleaned using in-house Perl scripts to remove reads containing adapter or poly-*N* sequences, as well as low-quality reads (i.e., those where the sequencing error rate was >0.1%). Clean reads were mapped to the *L. vannamei* genome using Hisat2 2.1.0^2^ (Kim et al., 2015). Unique mapped reads with one or zero mismatches were used to calculate gene expression levels.

We used the DESeq2 package (Love et al., 2014) to identify statistical differences in gene expression between samples. Genes were considered significantly differentially expressed when the false discovery rate (FDR) was <0.01 and fold change was ≥2.

The differentially expressed genes (DEGs) were annotated against the following databases: Nr^3^, GO^4^, and KEGG^5^.

### Candidate Gene Analysis

The DEG sequences were compared to the *L. vannamei* genomic region within each QTL using the NCBI blast tool^6^. We considered a DEG putatively associated with nitrite tolerance only if the DEG was located within the QTL interval, and the expression profile of the DEG was consistent across all four families (LV-1, LV-2, LV-3, and LV-4). Preliminary analysis identified a single gene meeting these criteria: *SLC26A6*.

### Verification of SLC26A6 Expression Using qRT-PCR

To validate our RNA-seq results, we used qRT-PCR to quantify the expression of the candidate gene (*SLC26A6*) in the nitrite-tolerant and nitrite-sensitive pooled samples from the four *L. vannamei* families (LV-1, LV-2, LV-3, and LV-4). qRT-PCRs and RNA-seq analyses were performed using the same samples. qRT-PCRs were performed with SYBR Premix Ex TaqTM II kits (TaKaRa, Japan), following the manufacturer’s instructions. The primers used to detect *SLC26A6* gene expression levels were RT-SLC-F and RT-SLC-R (Table 1). The qRT-PCR cycling program was as follows: preheating at 95°C for 30 s, followed by 40 cycles of 95°C for 5 s and 60°C for 30 s. We used *L. vannamei* 18S RNA as the internal reference gene; this gene was amplified using the primers 18s-F and 18s-R (Zhang et al., 2008) (Table 1). Three parallel qRT-PCRs were performed for each sample. Relative gene expression levels were calculated using the 2^–ΔΔCT^ method (Livak and Schmittgen, 2001).

**TABLE 1:** Primers used in this study.

Name	Sequence (5′–3′)
**Primers for quantitative real-time PCR**	
18s-F	CTCTGCTGAACCGCATTACTTG
18s-R	TGCCGAGGGTTTTGGTCT
RT-SLC26A6-F	AGATAGCGTTCGGTCTGCTG
RT-SLC26A6-R	GAGGGACTTGACCTGTGACG
**Primers for gene silencing**	
SLC26A6-F	GAGAAGCGAAGTTTGTGCG
SLC26A6-R	CGTCGTGAAGCCTGAGATG
T7-SLC26A6-F	GGATCCTAATACGACTCACTATAGGGAGAAGCGAAGTT TGTGCG
T7-SLC26A6-R	GGATCCTAATACGACTCACTATAGGCGTCGTGAAGCCT GAGATG
T7-egfp-F	GGATCCTAATACGACTCACTATAGGGTGCCCATCCTGGT CGAGCT
T7-egfp-R	GGATCCTAATACGACTCACTATAGGTGCACGCTGCCGTC CTCGAT
egfp-F	GTGCCCATCCTGGTCGAGCT
egfp-R	TGCACGCTGCCGTCCTCGAT

### Effects of Candidate Gene Silencing on Nitrite Tolerance

The dsRNA used to silence the candidate gene (dsRNA-*SLC26A6*) was synthesized with the T7 RiboMAX Express RNAi System kit (Promega, United States), following the manufacturer’s instructions, using the primers T7-SLC26A6-F, T7-SLC26A6-R, SLC26A6-F, and SLC26A6-R (Table 1). The primers T7-egfp-F, T7-egfp-R, egfp-F, and egfp-R (Table 1) were used to synthesize dsRNA-egfp.

The dsRNA interference experiment was performed in 1,000-liter glass saltwater tanks (30‰ salinity; 26–27°C; pH 7.5–8.1). We randomly selected 360 *L. vannamei* from family LV-1. The selected shrimp were divided into three groups (*n* = 40 shrimp per group; three replicates of each group): buffer control, negative control, and experimental. Each shrimp in the buffer control group was injected at the second abdominal segment with 20 μg 0.9% normal saline; each shrimp in the negative control group was injected with 20 μg dsRNA-egfp; and each shrimp in the experimental group was injected with 20 μg dsRNA-*SLC26A6*. Each shrimp was re-injected after 24 h. At 12 h after the second injection, all shrimp were subjected to the acute nitrite stress test, as described above, for 120 h. The time of death of each shrimp was recorded.

To evaluate the inhibitory effects of dsRNA on *SLC26A6* gene expression, we randomly collected shrimp from the experimental group at 0, 36, 48, and 72 h after the first injection of dsRNA-*SLC26A6* (three shrimp were collected at each time point). The hepatopancreases were removed from the sampled shrimp as described above, and pooled by time point. Total RNA was extracted from each pool using a TRIzol Reagent kit (Invitrogen, United States), following the manufacturer’s instructions. qRT-PCRs was performed using SYBR Premix Ex TaqTM II kits (TaKaRa, Japan), following the manufacturer’s instructions. The primers used to detect *SLC26A6* expression (SLC26A6-F and SLC26A6-R; Table 1) were designed using Primer Premier v 6.2^7^ based on the *SLC26A6* gene sequence (NCBI accession: XM_027371736.1) (Zhang et al., 2019). The qRT-PCR cycling program was as follows: preheating at 95°C for 30 s, followed by 40 cycles of 95°C for 5 s and 60°C for 30 s. We used *L. vannamei* 18S RNA as the internal reference gene; this gene was amplified using the primers 18s-F and 18s-R (Table 1). Three biological replicates and three technical replicates were performed for each group. Relative mRNA expression levels were calculated using the 2^–ΔΔCT^ method (Livak and Schmittgen, 2001). Data are shown as mean ± standard deviation (SD), and analyzed using one-way ANOVAs in SPSS 13.0 (Link and Pachaly, 1975). We considered *P* < 0.01 statistically significant.

## Results

### Assessment of Nitrite Tolerance in the Mapping Family

To measure nitrite tolerance, 157 shrimp from the *L. vannamei* mapping family (LV-1) were exposed to high concentrations of nitrite in seawater; survival time was recorded to indicate nitrite tolerance. All experimental shrimp died after 2–83 h of exposure. The average survival time was 43 h. Individual survival time varied widely and was normally distributed. Therefore, the nitrite-tolerance trait was suitable for QTL detection.

### Construction of a High-Density Genetic Linkage Map

We constructed *L. vannamei* SLAF libraries from the two parents and the 157 progeny tested for nitrate tolerance. High-throughput sequencing of the libraries yielded 262.12 gigabases (Gb) of data, consisting of 1,310.33 million base (Mb) paired-end reads, each 100 bp in length (Supplementary Table S3). On average, the percentage of bases with a sequencing quality value ≥30 (Q30) was 95.63%, while the average GC content was 40.31%. This indicated that the quality of the sequencing was good, and the GC distribution was normal. In total, 59.34% of the paired-end reads mapped successfully to the *L. vannamei* genome. We used the rice (*Oryza sativa japonica*) genome as a control to estimate the validity of our library construction. For the rice library, we generated 343.21 Mb of data (1.72 Mb paired-end reads), with a Q30 of 95.81% and a GC content of 40.96%. In rice, 91.43% of the paired-end reads were mapped successfully to the rice genome. These results indicated that SLAF library construction and sequencing were normal.

After filtering and clustering, 1,079,516 SLAF markers were identified, with an average sequencing depth of 48.99-fold for the male parent, 46.61-fold for the female parent, and 13.19-fold for the offspring. After further filtering, we identified 219,463 polymorphic SLAF markers. These polymorphic markers were genotyped, and 101,907 were successfully encoded into eight genotypes (ab × cd, ef × eg, hk × hk, lm × ll, nn × np, aa × bb, ab × cc, and cc × ab). As the mapping population was an F2 population, polymorphic markers with the aa × bb segregation pattern (the homozygous genotype) were discarded.

**FIGURE 1:**
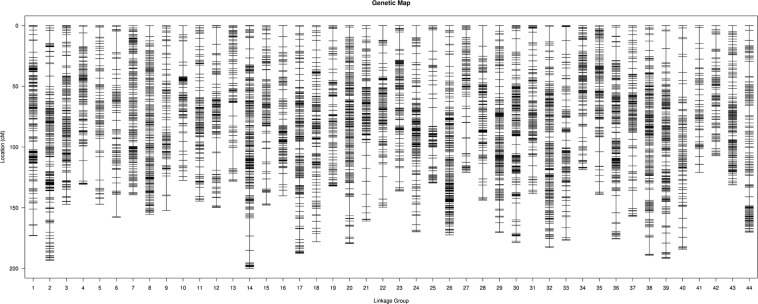
High-density linkage sex-average map for *Litopenaeus vannamei*, showing genetic distances among specific length amplified fragment (SLAF) markers (shown as black bars).

After analyzing the linkages among the remaining SLAF markers, three maps (male, female, and sex-average) were constructed. In total, 17,242 SLAF markers were labeled across all three genetic maps: 10,276 on the male map; 11,543 on the female map; and 17,242 on the sex-average map (Figure 1). Each of the three maps contained 44 LGs (Supplementary Tables S4–S6). The average genetic distance between markers on the male map was 0.60 cM (total genetic distance: 6,164.79 cM); the average genetic distance between markers on the female map was 0.60 cM (total genetic distance: 6,906.78 cM); and the average genetic distance between markers on the sex-average map was 0.4 cM (total genetic distance: 6,828.06 cM).

### QTL Mapping of Nitrite Tolerance

**FIGURE 2:**
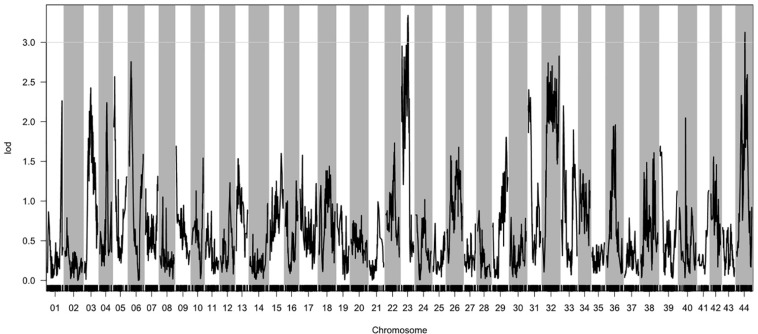
Quantitative trait loci (QTL) for nitrite tolerance in *Litopenaeus vannamei*, showing the logarithm of odds (LOD) values of the linkage groups. The gray line indicates the LOD threshold (3.00; *P* = 0.05).

Based on the genetic maps and the experimentally collected nitrite-tolerance data for the mapping family, we performed a QTL analysis of the nitrite-tolerance trait. The threshold LOD score, above which QTLs were considered significant, was determined to be 3.0 based on 1,000 permutations (*P* < 0.05). Using this threshold, we identified two QTLs (QNT1 and QNT2) for nitrite tolerance (Figure 2). The QTLs (LOD values: 3.00–3.35) were located on LG23 and LG44, with confidence intervals of 67.87–77.59 cM and 91.35–93.51 cM, respectively (Supplementary Table S7). The phenotypic variation explained by these QTLs was 8.42–10.31% (Table 2).

**TABLE 2:** Quantitative trait loci (QTLs) for nitrite tolerance detected in the mapping family (LV-1).

QTL	Linkage group	Interval of QTL (cM)	Logarithm of the odds (LOD)	Phenotypic variance explained (PVE) by QTL
QNT1	23	67.87–77.59	3.00–3.35	8.42%
QNT2	44	91.35–93.51	3.00–3.12	10.31%

### Transcriptomic Profiles of Nitrite-Tolerant and Nitrite-Sensitive Shrimp From Different Families

**FIGURE 3:**
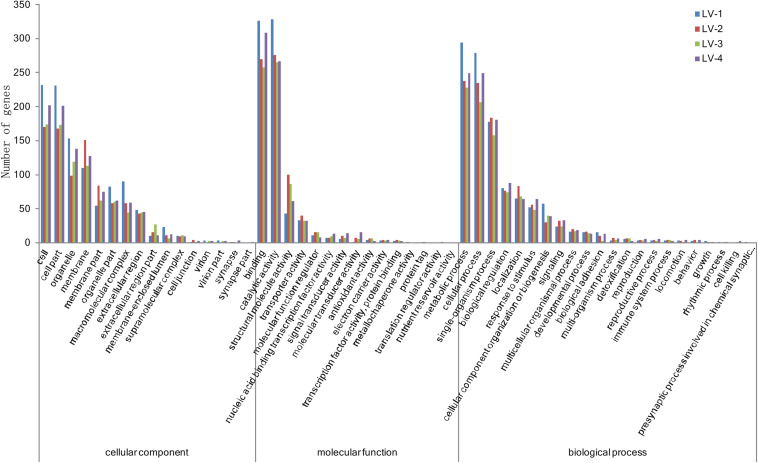
The Gene Ontology (GO) terms overrepresented in the differentially expressed genes (DEGs) across four genetically distinct *Litopenaeus vannamei* families (LV-1, LV-2, LV-3, and LV-4).

**FIGURE 4:**
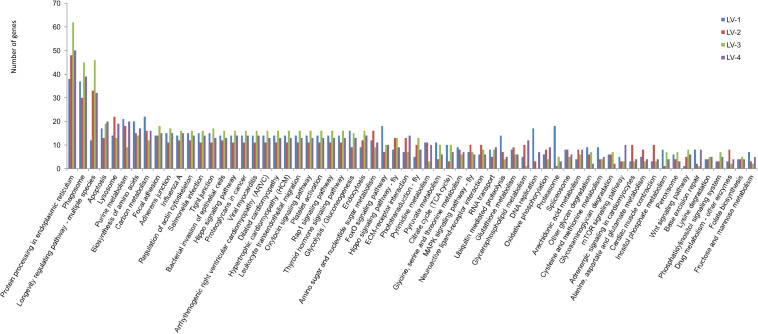
The KEGG terms overrepresented in the in the DEGs across four genetically distinct *Litopenaeus vannamei* families (LV-1, LV-2, LV-3, and LV-4).

To identify candidate genes associated with nitrite tolerance in *L. vannamei*, we generated eight cDNA libraries using mRNA extracted from the pooled hepatopancreases of the 20 most nitrite-tolerant and the most 20 nitrite-sensitive shrimp in four genetically distinct families. After quality control, a total of 58.18 Gb of clean data were obtained. Using these data, we identified 2,002, 1,983, 1,954, and 1,867 DEGs between the nitrite-tolerant and nitrite-sensitive shrimp in the families LV-1, LV-2, LV-3, and LV-4, respectively (Supplementary Table S8). The DEGs were annotated based on the databases Nr (non-redundant protein sequences), GO (Gene Ontology), and KEGG (see text footnote 5). Notably, the GO and KEGG terms overrepresented in the DEGs were similar across all four families. The most overrepresented GO terms in the DEGs were “cell,” “cell part,” “binding,” “catalytic activity,” “metabolic process,” “cellular process,” and “single-organism process” (Figure 3), while the most overrepresented KEGG terms were “Protein processing in endoplasmic reticulum,” “Phagosome” and “Longevity regulating pathway-multiple species” (Figure 4).

### Candidate Gene Analysis

**FIGURE 5:**
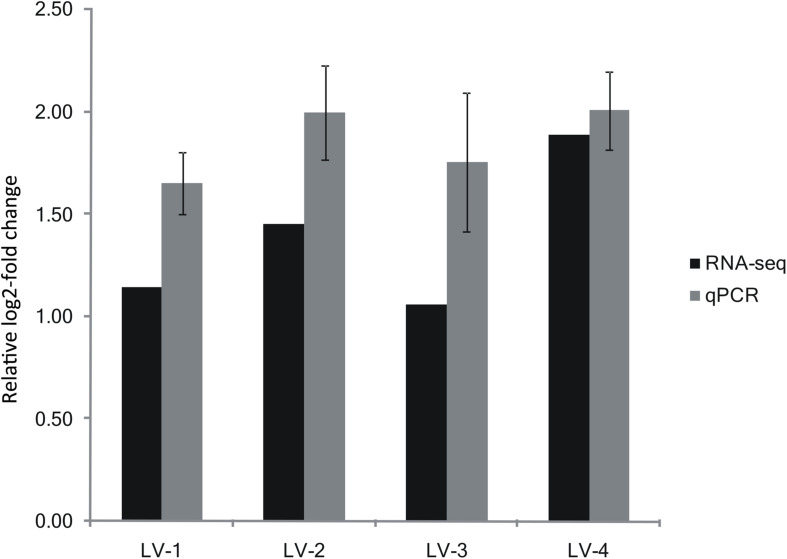
Expression of *SLC26A6* from the transcriptomic analysis validated by qRT-PCR. Expression of *SLC26A6* was detected in nitrite-sensitive and nitrite-tolerant shrimp from genetically distinct *Litopenaeus vannamei* families (LV-1, LV-2, LV-3, and LV-4). Data were normalized to 18s rRNA expression, and presented as a relative log2-fold change to validate the transcriptomic analysis results. Error bars show the standard deviation of three technical replicates.

Based on our alignment of the DEGs with the QTL regions in LG23 and LG44, we found that 231 DEGs were located in a QTL interval (the expression profiles and annotations of these DEGs are listed in Supplementary Table S8). Of these DEGs, only one gene (LOC113819511) exhibited a consistent pattern of expression between the nitrite-tolerant and nitrite-sensitive groups across all four families. This gene was annotated as solute carrier family 26 member 6 (*SLC26A6*; Zhang et al., 2019). *SLC26A6* was located within the QTL interval of LG23, and was significantly upregulated in the most nitrite-tolerant shrimp compared to the most nitrite-sensitive shrimp across all the four families (Supplementary Table S9). The log2-fold changes in *SLC26A6* gene expression level between the nitrite-sensitive and nitrite-tolerant shrimp were 1.14, 1.45, 1.06, and 1.89 in the LV-1, LV-2, LV-3, and LV-4 families, respectively (Figure 5). Thus, *SLC26A6* was considered a candidate gene associated with nitrite resistance.

### Verification of Candidate Gene Expression Using Quantitative Real-Time PCR (qRT-PCR)

The patterns of *SLC26A6* gene expression in nitrite-tolerant and nitrite-sensitive pooled samples from the families LV-1, LV-2, LV-3, and LV-4 were similar to the patterns determined using RNA-seq: *SLC26A6* gene expression was significantly greater in the nitrite-tolerant shrimp as compared to the nitrite-sensitive shrimp across all four families (Figure 5). The log2-fold changes in *SLC26A6* gene expression level between the nitrite-sensitive and nitrite-tolerant shrimp were 1.65, 1.99, 1.76, and 2.01 in the LV-1, LV-2, LV-3, and LV-4 families, respectively (Figure 5).

### Effects of Candidate Gene Silencing on Nitrite Tolerance in Shrimp

**FIGURE 6:**
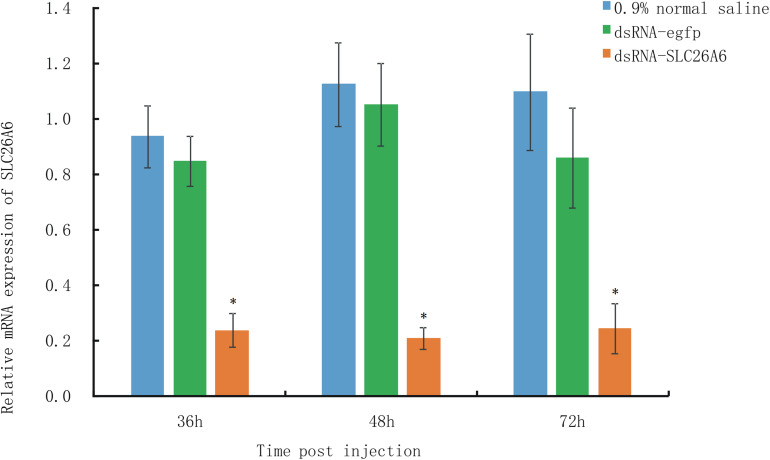
qRT-PCR quantification of relative *SLC26A6* expression in shrimp from *Litopenaeus vannamei* family LV-1 at 36, 48, and 72 h after injection with 0.9% normal saline (blue bars; buffer control), dsRNA-egfp (green bars; negative control), or dsRNA-*SLC26A6* (orange bars; experimental group). *L. vannamei* 18S RNA was used as the internal reference gene. Asterisks (*) above bars indicate significant differences (*P* < 0.01) in relative gene expression as compared to both controls. Error bars show the standard deviation of three biological replicates.

**FIGURE 7:**
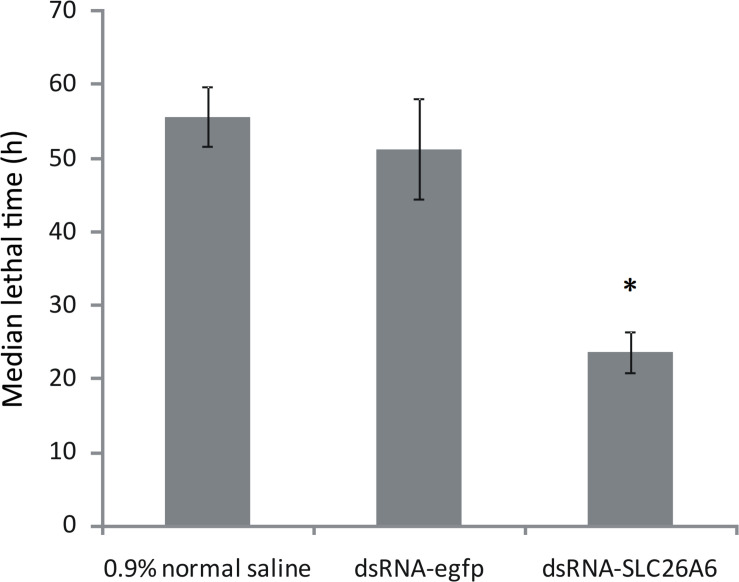
Median lethal time of *Litopenaeus vannamei* after injection with 0.9% normal saline (buffer control), dsRNA-egfp (negative control), or dsRNA-*SLC26A6* (experimental group). Asterisks (*) above bars indicate significant differences (*P* < 0.01) in median lethal time as compared to both controls. Error bars show the standard deviation of three biological replicates.

qRT-PCR analysis showed that in shrimp collected 36, 48, and 72 h after the injection of dsRNA, *SLC26A6* mRNA expression was significantly (*p* < 0.01) inhibited in comparison to the buffer control group (injected with saline) and the negative control group (injected with dsRNA-egfp; Figure 6). The median lethal time (LT50) of shrimp injected with dsRNA-*SLC26A6* was 23.67 ± 2.87 h, which was significantly shorter than that of shrimp injected with dsRNA-egfp (51.33 ± 6.94 h) and that of shrimp injected with normal saline (55.67 ± 4.03 h) (Figure 7). This indicated that the silencing of *SLC26A6* gene expression significantly reduced nitrite tolerance in *L. vannamei*.

## Discussion

Although genetic maps for *L. vannamei* have been previously published, most of these were generated using AFLP or SSR markers alone or in combination with other markers (Abdelrahman et al., 2017). In such genetic maps, the number of mapped markers is relatively small, and the average distance between adjacent markers is relatively long. For example, Zhang et al. (2007) constructed a genetic linkage map of *L. vannamei* using 816 AFLP and SSR markers; the average density of this map was 15.1 cM. Similarly, Andriantahina et al. (2013) constructed a genetic map of *L. vannamei* using 451 AFLP and SSR markers; the average density of this map was 7.6 cM. Here, we constructed a genetic linkage map of *L. vannamei* using 17,242 SLAF markers mapped to 44 LGs; the average density of this map was 0.40 cM. To our knowledge only one other study has used SLAF markers to create a genetic map of *L. vannamei*, but this previous study included only 6,146 SNP markers, with an average distance of 0.7 cM (Yu et al., 2015). Therefore, our genetic map contained more markers and spanned longer genetic distances than previously available high-density genetic maps of *L. vannamei*. This may be because the sequencing depth in this study was greater than that in previous studies.

We used these high-density genetic maps to investigate genes associated with nitrite tolerance in *L. vannamei*. Nitrite is one of the most common contaminants of aquaculture systems. Nitrite pollution has deleterious effects on shrimp, inhibiting growth and immunity, stimulating stress responses, and increasing disease risks; in some cases, high nitrite levels may even cause shrimp death (Tseng and Chen, 2004; Brown et al., 2013; Guo et al., 2016; Duan et al., 2018; Valencia-Castaneda et al., 2018).

As shrimp are an important aquaculture organism, it is therefore critical to identify the genetic mechanisms underlying nitrite tolerance in shrimp. To this end, we used our genetic map to identify two QTLs for nitrite tolerance in *L. vannamei*, located on LG23 and LG44 (LOD values: 3.00–3.71; confidence intervals: 67.87–77.59 cM and 91.35–93.51 cM, respectively); these QTLs explained 8.42–10.31% of the phenotypic variation. To the best of our knowledge, this is the first identification of QTLs for nitrite tolerance in *L. vannamei*. The identification of these QTLs will support further studies aiming to develop new, nitrite-tolerant varieties of *L. vannamei*. However, the proportion of genetic variation explained by the markers discovered in this article is not large enough to propose use them in breeding. Therefore in future research, we should relaxe the cutoff of LOD score to get more QTLs, intervals, and candidate DEGs. And then we should identify more candidate genes associated with nitrite tolerance.

Quantitative trait loci analyses based on high-density genetic maps are commonly used to identify candidate genes associated with specific traits of interest (Cervino et al., 2005). However, QTL regions typically contain several hundred genes (Wayne and McIntyre, 2002). Comprehensive analysis of gene expression profiles can be effectively used in conjunction with QTLs to reduce the number of potential candidate genes. We thus performed RNA-seq analyses of four genetically distinct *L. vannamei* families to identify candidate genes associated with nitrite-tolerance. We identified 7,806 genes that were differentially expressed between the nitrite-tolerant and nitrite-sensitive shrimp across all four families; of these, only a single gene, SLC26A6, was located in a QTL interval and consistently exhibited the same expression pattern irrespective of family. In RNA-seq and qRT-PCR analyses of all four families, SLC26A6 was upregulated in nitrite-tolerant shrimp, and downregulated in nitrite-sensitive shrimp. Consistent with these results, we found that, when SLC26A6 gene expression was silenced using dsRNA, shrimp LT50 during acute nitrite exposure decreased significantly as compared to unsilenced controls. This suggested that SLC26A6 expression was essential for nitrite tolerance in shrimp. SLC26A6 belongs to the ten-member SLC26 gene family; genes in this family encode anion exchangers and are able to transport a wide variety of monovalent and divalent anions (Mount and Romero, 2004). Numerous studies have demonstrated that the SLC26A6 protein is expressed on the cell membrane and acts as an important Cl^–^/HCO3^–^ exchanger (Jiang et al., 2006; Niederer et al., 2008). Some studies have shown that upregulation of SLC26A6 promotes Cl^–^ absorption and HCO3^–^ excretion in aquatic animal cells (Kurita et al., 2008; Grosell et al., 2009; Boyle et al., 2015; Ping et al., 2015; Gerber et al., 2018). For example, a previous study found that the slc26a6 gene of the nakedcarp (*Gymnocypris przewalskii*) was expressed in various tissues, such as gills, liver, kidneys and intestines, and that the upregulation of SLC26A6 increased HCO3^–^ secretion and the Cl^–^ absorption in the cells (Ping et al., 2015). Notably, several studies have shown that elevated chloride concentrations in the aquatic environment reduce the toxic effects of nitrite on aquatic animals, because both nitrite ions and chloride ions compete for the same site of active transport (Alcaraz and Espina, 1994; Jiann-Chu and Lee, 1997; Sha-Yen and Chen, 1998; Yildiz and Benli, 2004; Kozák et al., 2005; Camargo and Alonso, 2006; Alonso and Camargo, 2008; Zhang et al., 2015). Therefore, we hypothesized that the upregulation of SLC26A6 in nitrite-tolerant shrimp increases the absorption of chloride ions, forcing chloride ions and nitrite ions compete for the same site of active transport on the plasma membrane and various organelle membranes, resulting in enhanced nitrite tolerance. Thus, SLC26A6 may be a useful a candidate gene associated with nitrite tolerance in *L. vannamei*. However, the detailed mechanisms underlying these effects require further study.

To date, there are few studies on nitrite tolerance in aquatic animals. A single recent study investigated the physiology, transcriptome, and metabolome of *L. vannamei*, and found that nitrite tolerance in *L. vannamei* might be associated with the upregulation of hypoxia inducible factor-1α to regulate energy supply and gaseous exchange (Xiao et al., 2020). However, our results did not locate hypoxia inducible factor-1α in the identified QTL regions. This may might because the two studies used different shrimp families. Alternatively, there may be multiple mechanisms underlying nitrite tolerance in shrimp; this possibility requires further research.

## Conclusion

We used the SLAF-seq method to construct high-density genetic maps of *L. vannamei*, and to identify QTLs associated with nitrite tolerance. By combining QTL and transcriptome analyses, we identified a candidate gene associated with nitrite tolerance. Our work increases our understanding of the molecular mechanisms underlying nitrite tolerance in shrimp and provides a basis for molecular-marker-assisted shrimp breeding.

## Data Availability Statement

The datasets generated for this study can be found in the Genebank accession numbers: PRJNA545877, SRR9822088, SRR9822087, SRR9822086, SRR9822092, SRR9822089, SRR9822096, SRR9822110, and SRR9822097.

## Author Contributions

YZ and XHC conceived the study. DZ and XLC wrote the manuscript and contributed to the bioinformatics analysis. WZ, MP, and XHC conducted the experiments and contributed to the raw data analysis. QLiu, QLi, YL, HW, HL, and JL contributed to the bioinformatics analysis. All authors read and approved the final manuscript.

## Conflict of Interest

The authors declare that the research was conducted in the absence of any commercial or financial relationships that could be construed as a potential conflict of interest.

## Footnotes

1 https://www.ncbi.nlm.nih.gov/genome/?term=Vannamei

2 http://ccb.jhu.edu/software/hisat2/index.shtml

3 ftp://ftp.ncbi.nih.gov/blast/db/

4 http://www.geneontology.org/

5 http://www.genome.jp/kegg/

6 https://blast.ncbi.nlm.nih.gov/Blast.cgi

7 http://premierbiosoft.com/primerdesign/index.html
