# Supplementary material for: Construction of a High-Density Genetic Map and Identification of Quantitative Trait Loci for Nitrite Tolerance in the Pacific White Shrimp (Litopenaeus vannamei)
Source: Front Genet. 2020 Sep 24;11:571880. doi: 10.3389/fgene.2020.571880 (PMC7541944; doi:10.3389/fgene.2020.571880)
Supplement: Supplementary file 1 [file Table_1.DOCX]

**Supplementary table S1.** Acute nitrite stress test for *Litopenaeus vannamei* at different nitrite concentration.

| Nitrite concentration (mg/l) | Number of shrimp | Earliest death time (h) | Latest lethal time (h) | Median lethal time (h) | Average lethal time (h) |
| --- | --- | --- | --- | --- | --- |
| 500 | 40 | 34 | 192 | 143 | 124 |
| 600 | 40 | 18 | 137 | 88 | 75 |
| 700 | 40 | 4 | 80 | 51 | 41 |
| 800 | 40 | 1 | 52 | 32 | 29 |

Note: pH maintained at 8.2 ± 0.3; temperature maintained at 27.0 ± 0.5°C; salinity maintained at 30.1‰; and dissolved oxygen maintained at 7–8 mg/L.
