# Supplementary material for: Construction of a High-Density Genetic Map and Identification of Quantitative Trait Loci for Nitrite Tolerance in the Pacific White Shrimp (Litopenaeus vannamei)
Source: Front Genet. 2020 Sep 24;11:571880. doi: 10.3389/fgene.2020.571880 (PMC7541944; doi:10.3389/fgene.2020.571880)
Supplement: Supplementary file 2 [file Table_2.DOCX]

**Supplementary Table S2.** Median lethal concentration (LC50) of NaNO_2_ for *Litopenaeus vannamei* families LV-1, LV-2, LV-3, and LV-4.

| Family | 24h-LC50（mg/L） | 48h-LC50（mg/L） | 72h-LC50（mg/L） | 96h-LC50（mg/L） |
| --- | --- | --- | --- | --- |
| LV-1 | 194.611 | 131.507 | 108.105 | 95.392 |
| LV-2 | 196.345 | 161.825 | 124.878 | 106.488 |
| LV-3 | 174.374 | 61.857 | 40.240 | 23.133 |
| LV-4 | 250.821 | 160.421 | 125.945 | 94.741 |

Note: pH maintained at 8.2 ± 0.3; temperature maintained at 27.0 ± 0.5°C; salinity maintained at 30.1‰; and dissolved oxygen maintained at 7–8 mg/L.
