# Supplementary material for: Construction of a High-Density Genetic Map and Identification of Quantitative Trait Loci for Nitrite Tolerance in the Pacific White Shrimp (Litopenaeus vannamei)
Source: Front Genet. 2020 Sep 24;11:571880. doi: 10.3389/fgene.2020.571880 (PMC7541944; doi:10.3389/fgene.2020.571880)
Supplement: Supplementary file 3 [file Table_3.DOCX]

**Supplementary table S3.** Summary of the constructed genetic map of *Litopenaeus vannamei*.

| Subject | Value |
| --- | --- |
| Total bases | 262.12 Gb |
| Total reads | 1,310.33 Mb |
| Average Q30 | 95.63% |
| Average GC | 40.31% |
| Enzyme digestion protocol | HaeIII+Hpy166II |
| Restriction fragment length | 314-414 bp |
| Percentage of reads matching the *L. vannamei* genome | 59.34% |
| Number of high-quality SLAFs | 1,079,516 |
| Number of polymorphic SLAFs | 219,463 |
| Number of SLAF markers on the map | 17,242 |
| Average sequencing depth of SLAF for female parent | 48.99 × |
| Average sequencing depth of SLAF for male parent | 46.61× |
| Average sequencing depth of SLAF for offspring individual | 13.19 × |
| Number of linkage groups | 44 |
| Total distance of the map | 6,828.06 cM |
| Average distance of the map | 0.40 cM |
