# Supplementary material for: Construction of a High-Density Genetic Map and Identification of Quantitative Trait Loci for Nitrite Tolerance in the Pacific White Shrimp (Litopenaeus vannamei)
Source: Front Genet. 2020 Sep 24;11:571880. doi: 10.3389/fgene.2020.571880 (PMC7541944; doi:10.3389/fgene.2020.571880)
Supplement: Supplementary file 4 [file Table_4.DOCX]

**Supplementary table S4.** Basic information for the male map.

| Linkage | Total | Total | Average | Max | Gap |
| --- | --- | --- | --- | --- | --- |
| Group ID | Marker | Distance(cM) | Distance(cM) | Gap (cM) | < 5 cM（%） |
| 1 | 357 | 158.76 | 0.45 | 18.25 | 98.31 |
| 2 | 451 | 186.16 | 0.41 | 13.03 | 97.56 |
| 3 | 248 | 141.89 | 0.57 | 11.97 | 97.57 |
| 4 | 212 | 127.03 | 0.6 | 48.94 | 99.05 |
| 5 | 119 | 178.47 | 1.51 | 67.13 | 94.07 |
| 6 | 187 | 142.37 | 0.77 | 36.94 | 98.39 |
| 7 | 202 | 183.84 | 0.91 | 6.81 | 98.01 |
| 8 | 261 | 157.29 | 0.6 | 5.27 | 99.62 |
| 9 | 157 | 138.76 | 0.89 | 15.68 | 95.51 |
| 10 | 229 | 81.22 | 0.36 | 9.82 | 97.81 |
| 11 | 218 | 116.85 | 0.54 | 9.5 | 97.7 |
| 12 | 167 | 157 | 0.95 | 27.91 | 95.78 |
| 13 | 149 | 56.73 | 0.38 | 5.37 | 99.32 |
| 14 | 401 | 183.11 | 0.46 | 23.85 | 98.75 |
| 15 | 184 | 166.86 | 0.91 | 19.07 | 96.17 |
| 16 | 265 | 117.67 | 0.45 | 13.86 | 97.73 |
| 17 | 397 | 190.96 | 0.48 | 26.12 | 98.23 |
| 18 | 298 | 172.93 | 0.58 | 20.11 | 96.63 |
| 19 | 220 | 123.13 | 0.56 | 23.06 | 97.26 |
| 20 | 393 | 184.86 | 0.47 | 24 | 98.98 |
| 21 | 367 | 142.58 | 0.39 | 38.29 | 98.63 |
| 22 | 185 | 116.07 | 0.63 | 18.25 | 95.65 |
| 23 | 76 | 90.46 | 1.21 | 18.04 | 93.33 |
| 24 | 461 | 161.26 | 0.35 | 22.06 | 98.48 |
| 25 | 204 | 135.08 | 0.67 | 33.09 | 97.54 |
| 26 | 501 | 148.86 | 0.3 | 9.82 | 99.2 |
| 27 | 202 | 107.97 | 0.54 | 16.22 | 98.01 |
| 28 | 246 | 120.11 | 0.49 | 48.94 | 98.37 |
| 29 | 293 | 163.69 | 0.56 | 12.21 | 96.92 |
| 30 | 349 | 176.43 | 0.51 | 23.46 | 97.99 |
| 31 | 194 | 145.96 | 0.76 | 21.08 | 96.37 |
| 32 | 336 | 134.82 | 0.4 | 6.5 | 99.4 |
| 33 | 123 | 136.11 | 1.12 | 44.09 | 95.08 |
| 34 | 122 | 91.95 | 0.76 | 11.16 | 95.87 |
| 35 | 32 | 82.11 | 2.65 | 45.66 | 83.87 |
| 36 | 204 | 120.71 | 0.59 | 17.86 | 97.54 |
| 37 | 279 | 170.27 | 0.61 | 29.52 | 97.48 |
| 38 | 225 | 180.3 | 0.8 | 26.18 | 96.43 |
| 39 | 136 | 167.19 | 1.24 | 31.83 | 94.81 |
| 40 | 53 | 134.53 | 2.59 | 41.11 | 86.54 |
| 41 | 50 | 135.11 | 2.76 | 16.86 | 77.55 |
| 42 | 114 | 77.34 | 0.68 | 6.7 | 97.35 |
| 43 | 242 | 133.89 | 0.56 | 5.38 | 98.76 |
| 44 | 167 | 126.1 | 0.76 | 20.11 | 96.99 |
| Total | 10,276 | 6,164.79 | 0.6 | 67.13 | 77.55 |
