# Supplementary material for: Construction of a High-Density Genetic Map and Identification of Quantitative Trait Loci for Nitrite Tolerance in the Pacific White Shrimp (Litopenaeus vannamei)
Source: Front Genet. 2020 Sep 24;11:571880. doi: 10.3389/fgene.2020.571880 (PMC7541944; doi:10.3389/fgene.2020.571880)
Supplement: Supplementary file 5 [file Table_5.DOCX]

**Supplementary table** S5. Basic information for the female map.

| Linkage | Total | Total | Average | Max | Gap |
| --- | --- | --- | --- | --- | --- |
| Group ID | Marker | Distance(cM) | Distance(cM) | Gap (cM) | < 5 cM（%） |
| 1 | 395 | 187.42 | 0.48 | 13.02 | 97.97 |
| 2 | 482 | 185.96 | 0.39 | 24.08 | 98.75 |
| 3 | 246 | 152.48 | 0.62 | 23.06 | 97.96 |
| 4 | 206 | 128.85 | 0.63 | 13.86 | 97.07 |
| 5 | 57 | 80.57 | 1.44 | 34.34 | 92.86 |
| 6 | 148 | 172.62 | 1.17 | 45.66 | 95.24 |
| 7 | 183 | 83.49 | 0.46 | 14.7 | 98.35 |
| 8 | 175 | 141.14 | 0.81 | 21.08 | 97.7 |
| 9 | 142 | 137.48 | 0.98 | 26.18 | 96.45 |
| 10 | 262 | 128.75 | 0.49 | 17.34 | 96.55 |
| 11 | 208 | 159.04 | 0.77 | 24.6 | 95.65 |
| 12 | 180 | 142.49 | 0.8 | 17.31 | 96.09 |
| 13 | 147 | 134.3 | 0.92 | 26.18 | 94.52 |
| 14 | 388 | 216.8 | 0.56 | 42.58 | 98.45 |
| 15 | 152 | 127.54 | 0.84 | 39.68 | 98.01 |
| 16 | 181 | 116.44 | 0.65 | 9.05 | 97.22 |
| 17 | 389 | 177.26 | 0.46 | 14.7 | 98.45 |
| 18 | 459 | 176.26 | 0.38 | 14.7 | 98.25 |
| 19 | 212 | 141.26 | 0.67 | 17.34 | 96.21 |
| 20 | 330 | 159.33 | 0.48 | 10.46 | 98.48 |
| 21 | 349 | 178.06 | 0.51 | 75.04 | 98.56 |
| 22 | 222 | 181.73 | 0.82 | 36.37 | 97.74 |
| 23 | 260 | 181.99 | 0.7 | 27.73 | 96.53 |
| 24 | 428 | 162.67 | 0.38 | 16.44 | 98.59 |
| 25 | 121 | 124.22 | 1.04 | 88.02 | 99.17 |
| 26 | 530 | 144.93 | 0.27 | 7.55 | 99.43 |
| 27 | 85 | 131.39 | 1.56 | 36.17 | 94.05 |
| 28 | 301 | 150.46 | 0.5 | 35.62 | 98.67 |
| 29 | 318 | 147.21 | 0.46 | 7.55 | 98.74 |
| 30 | 346 | 180.93 | 0.52 | 13.02 | 97.39 |
| 31 | 187 | 130.8 | 0.7 | 27.27 | 99.46 |
| 32 | 365 | 209.74 | 0.58 | 15.57 | 97.53 |
| 33 | 218 | 217.42 | 1 | 15.56 | 94.01 |
| 34 | 300 | 143.97 | 0.48 | 8.29 | 98.33 |
| 35 | 205 | 193 | 0.95 | 21.94 | 98.53 |
| 36 | 486 | 221.84 | 0.46 | 23 | 98.35 |
| 37 | 225 | 142.9 | 0.64 | 29.52 | 97.32 |
| 38 | 475 | 195.54 | 0.41 | 12.75 | 98.52 |
| 39 | 348 | 215.85 | 0.62 | 15.16 | 98.27 |
| 40 | 344 | 210.11 | 0.61 | 23.06 | 96.79 |
| 41 | 60 | 85.78 | 1.45 | 26.07 | 93.22 |
| 42 | 117 | 91.17 | 0.79 | 12.15 | 96.55 |
| 43 | 75 | 118.66 | 1.6 | 51.45 | 93.24 |
| 44 | 236 | 196.93 | 0.84 | 29.52 | 96.17 |
| Total | 11,543 | 6,906.78 | 0.6 | 88.02 | 92.86 |
