# Supplementary material for: Construction of a High-Density Genetic Map and Identification of Quantitative Trait Loci for Nitrite Tolerance in the Pacific White Shrimp (Litopenaeus vannamei)
Source: Front Genet. 2020 Sep 24;11:571880. doi: 10.3389/fgene.2020.571880 (PMC7541944; doi:10.3389/fgene.2020.571880)
Supplement: Supplementary file 6 [file Table_6.DOCX]

**Supplementary table S6.** Basic information for the sex-average map.

| Linkage | Total | Total | Average | Max | Gap |
| --- | --- | --- | --- | --- | --- |
| Group ID | Marker | Distance(cM) | Distance(cM) | Gap (cM) | < 5 cM（%） |
| 1 | 550 | 173.09 | 0.32 | 12.72 | 98.91 |
| 2 | 728 | 193.38 | 0.27 | 10.74 | 99.04 |
| 3 | 377 | 147.18 | 0.39 | 8.53 | 98.67 |
| 4 | 310 | 130.95 | 0.42 | 16.86 | 98.71 |
| 5 | 168 | 147.14 | 0.88 | 19.24 | 95.81 |
| 6 | 282 | 157.83 | 0.56 | 18.47 | 97.15 |
| 7 | 338 | 139.35 | 0.41 | 4.67 | 100 |
| 8 | 346 | 155.53 | 0.45 | 9.05 | 99.71 |
| 9 | 217 | 152.38 | 0.71 | 15.67 | 97.22 |
| 10 | 384 | 127.51 | 0.33 | 17.34 | 98.69 |
| 11 | 351 | 144.57 | 0.41 | 7.55 | 98 |
| 12 | 266 | 149.75 | 0.57 | 14.34 | 97.36 |
| 13 | 272 | 128.15 | 0.47 | 17.21 | 98.52 |
| 14 | 583 | 199.96 | 0.34 | 14.86 | 98.8 |
| 15 | 232 | 147.84 | 0.64 | 11.15 | 96.97 |
| 16 | 349 | 140.2 | 0.4 | 9.05 | 98.28 |
| 17 | 596 | 187.79 | 0.32 | 8.9 | 98.66 |
| 18 | 601 | 178 | 0.3 | 7.94 | 99.17 |
| 19 | 308 | 132.2 | 0.43 | 10.23 | 98.37 |
| 20 | 532 | 179.46 | 0.34 | 15.81 | 99.25 |
| 21 | 500 | 160.96 | 0.32 | 10.69 | 98.2 |
| 22 | 330 | 149.55 | 0.45 | 18.43 | 98.18 |
| 23 | 303 | 136.23 | 0.45 | 14.25 | 99.01 |
| 24 | 678 | 169.86 | 0.25 | 9.88 | 99.26 |
| 25 | 265 | 129.65 | 0.49 | 17.52 | 96.97 |
| 26 | 803 | 172.34 | 0.21 | 9.82 | 99.5 |
| 27 | 226 | 120.97 | 0.54 | 12.57 | 97.33 |
| 28 | 443 | 143.77 | 0.33 | 16.98 | 98.87 |
| 29 | 476 | 170.5 | 0.36 | 7.55 | 98.74 |
| 30 | 524 | 178.68 | 0.34 | 13.91 | 98.66 |
| 31 | 299 | 138.38 | 0.46 | 9.33 | 98.99 |
| 32 | 534 | 182.76 | 0.34 | 12.87 | 99.25 |
| 33 | 239 | 176.77 | 0.74 | 11.34 | 95.38 |
| 34 | 355 | 118.61 | 0.34 | 9.38 | 99.15 |
| 35 | 221 | 139.17 | 0.63 | 12.2 | 97.73 |
| 36 | 586 | 175.66 | 0.3 | 14.23 | 99.15 |
| 37 | 405 | 157.23 | 0.39 | 15.05 | 98.27 |
| 38 | 580 | 189.21 | 0.33 | 14.06 | 98.96 |
| 39 | 416 | 191.84 | 0.46 | 14.54 | 98.8 |
| 40 | 379 | 184.11 | 0.49 | 13.05 | 97.35 |
| 41 | 88 | 120.99 | 1.39 | 13.07 | 91.95 |
| 42 | 197 | 107.2 | 0.55 | 12.15 | 97.96 |
| 43 | 287 | 131.24 | 0.46 | 5.27 | 99.65 |
| 44 | 318 | 170.12 | 0.54 | 12.31 | 98.11 |
| Total | 17,242 | 6,828.06 | 0.4 | 19.24 | 91.95 |
