# Supplementary material for: Construction of a High-Density Genetic Map and Identification of Quantitative Trait Loci for Nitrite Tolerance in the Pacific White Shrimp (Litopenaeus vannamei)
Source: Front Genet. 2020 Sep 24;11:571880. doi: 10.3389/fgene.2020.571880 (PMC7541944; doi:10.3389/fgene.2020.571880)
Supplement: Supplementary file 8 [file Table_8.DOCX]

**Supplementary Table S8.** Number of differentially expressed genes (DEGs) identified in the *Litopenaeus vannamei* families LV-1, LV-2, LV-3, and LV-4.

| Family | DEG Set | DEG Number | Up-Regulated DEG | Down-Regulated DEG |
| --- | --- | --- | --- | --- |
| LV-1 | LV-1-NS / LV-1-NT | 2002 | 970 | 1032 |
| LV-2 | LV-2-NS / LV-2-NT | 1983 | 996 | 987 |
| LV-3 | LV-3-NS / LV-3-NT | 1954 | 1400 | 554 |
| LV-4 | LV-4-NS / LV-4-NT | 1867 | 775 | 1092 |
